# Exploring the Antimicrobial and Immunomodulatory Potential of Gecko-Derived Cathelicidin Gj-CATH5

**DOI:** 10.3390/biom15070908

**Published:** 2025-06-20

**Authors:** Shasha Cai, Ningyang Gao, Junhan Wang, Jing Li

**Affiliations:** The Key Laboratory for Medical Functional Nanomaterials, College of Medical Engineering, Jining Medical University, Jining 272067, China; gaoningyang23@gmail.com (N.G.); junhanwang548@gmail.com (J.W.)

**Keywords:** *Gekko japonicus*, cathelicidin, antimicrobial peptide, immunoregulation, *Pseudomonas aeruginosa*, anti-infection

## Abstract

Regulating the innate immune response against infections, particularly drug-resistant bacteria, is a key focus in anti-infection therapy. Cathelicidins, found in vertebrates, are crucial for pathogen resistance. Few studies have explored gecko cathelicidins’ anti-infection properties. Recently, five new cathelicidins (Gj-CATH1-5) were identified in *Gekko japonicus*. The peptide Gj-CATH5, from *G. japonicus*, shows promise against *Pseudomonas aeruginosa* through various mechanisms. This study examined Gj-CATH5’s protective effects using in vitro and in vivo models, finding that it significantly reduced bacterial load in a mouse infection model when administered before or shortly after infection. Flow cytometry and the plate counting method showed that Gj-CATH5 boosts neutrophil and macrophage activity, enhancing chemotaxis, phagocytosis, and bactericidal functions. Gj-CATH5 increases ROS production, MPO activity, and NET formation, aiding pathogen clearance. Its amphipathic α-helical structure supports broad-spectrum bactericidal activity (MBC: 4–8 μg/mL) against Gram-negative and antibiotic-resistant bacteria. Gj-CATH5 is minimally cytotoxic (<8% hemolysis at 200 μg/mL) and preserves cell viability at therapeutic levels. These results highlight Gj-CATH5’s dual role in pathogen elimination and immune modulation, offering a promising approach to combat multidrug-resistant infections while reducing inflammation. This study enhances the understanding of reptilian cathelicidins and lays the groundwork for peptide-based immune therapies against difficult bacterial infections.

## 1. Introduction

The efficacy of selectively activating or inhibiting the body’s immune response based on specific requirements has been demonstrated as an effective therapeutic approach, encompassing the prevention and treatment of infections, suppression of autoimmune diseases and inflammation, and stimulation of anti-tumor immune response in cancer treatment [1]. Presently, immunoregulatory anti-infection therapy primarily relies on the utilization of naturally occurring immune regulatory factors produced by the host immune system to rectify acquired or congenital immune deficiencies. The utilization of immunomodulation as a means of anti-infection therapy presents notable benefits in comparison to traditional antibiotics. The effective implementation of immunomodulatory anti-infection therapy necessitates meticulous regulation of the activation of the body’s defensive immune response, while concurrently minimizing the likelihood of detrimental inflammatory reactions. This approach is regarded as a potentially effective novel therapy for combating infections [2].

Cathelicidin is one group of IDRs that has been extensively studied in the context of its immunomodulatory activity. In recent years, as a result of the escalating challenges encountered in the research and development of novel small-molecule drugs with innovative chemical structures, coupled with the rising investment costs, researchers have progressively shifted their focus towards the advancement of polypeptide drugs [3]. In addition to its inherent bactericidal, fungicidal, virucidal, and protozoacidal properties [4,5], cathelicidin exhibits a diverse range of immunomodulatory activities, encompassing the direct or indirect stimulation of cell chemotaxis, regulation of dendritic cells and macrophages differentiation, promotion of angiogenesis and wound healing, activation of the acquired immune response, etc. [6,7]. As a new type of anti-infection reagent, cathelicidins have received extensive attention from scholars in China and abroad.

Relevant research has demonstrated that reptilian cathelicidins possess robust and extensive antibacterial properties, as well as high stability, low cytotoxicity, and potent immunomodulatory activity. These attributes make them a promising foundation for the development of anti-infection medications [8,9,10]. However, the majority of existing literature on reptilian cathelicidins has primarily focused on Serpentes and Crocodiliformes, such as king cobras, Burmese pythons, and Chinese alligators. Limited attention has been given to the cathelicidin of lacertiformes, specifically green lizards within the lizard family. Furthermore, studies have rarely been conducted on the anti-infection activity of cathelicidin within the gecko family. The medicinal use of geckos in China has a longstanding history and has been demonstrated to effectively treat various infectious diseases, including ulcers, carbuncles, bedsores, tuberculosis, and enteritis. However, there is currently a lack of research investigating the specific anti-infection and anti-inflammatory properties of geckos, as well as the underlying mechanisms of action.

In a previous study, a total of five novel cathelicidins (Gj-CATH5) were identified from *G. japonicus*. Notably, Gj-CATH2 demonstrated significant bactericidal and anti-biofilm properties against *Streptococcus mutans* [11]. Gj-CATH3 exhibited remarkable effectiveness in wound healing [12]. However, the biological activity of Gj-CATH5 remains insufficiently characterized. *Pseudomonas aeruginosa* is an opportunistic human pathogen, particularly in individuals with compromised immune systems and those suffering from cystic fibrosis. This bacterium’s resistance mechanisms and its impact on the immune response, such as the modulation of polymorphonuclear neutrophils (PMNs) and alveolar macrophages, make it a prime candidate for exploring innovative treatments that can enhance host immunity and bacterial clearance [13,14]. Therefore, the present study aims to investigate the in vitro and in vivo anti-infection activity of Gj-CATH5 against *P. aeruginosa*, as well as explore its potential mechanisms of action. This study would not only enhance our comprehension of the biological functions of cathelicidin but also hold significant implications for mitigating the issue of multidrug resistance among microorganisms and enhancing public health safety.

## 2. Materials and Methods

### 2.1. Peptides and Cells

Gj-CATH5 peptides (TRSRWRRFWGKAKRGIKKHGVSIALAAL RLRG) were synthesized by GL Biochemistry using a solid-phase peptide synthesis protocol based on Fmoc chemistry. High-performance liquid chromatography (HPLC) and electronic spray ionization-mass spectrometry (ESI−MS) were used to confirm a purity of >95.00%. The peptide was dissolved in sterile deionized H_2_O for further use. 

Primary mouse bone marrow-derived neutrophils (PMN) were isolated from the bone marrow of BALB/c mice using an animal bone neutrophil isolation kit (Solarbio Science & Technology, Beijing, China). The composition of the samples was confirmed to be >95% neutrophils through Wright–Giemsa staining. The viability of neutrophils was assessed by trypan blue. Cells were resuspended in RPMI-1640 medium supplemented with 10% FCS. Murine macrophage cell line J774A.1 was purchased from the American Type Culture Collection.

### 2.2. Ethics Statement

All animal experiments adhered to the Guidelines for the Care and Use of Laboratory Animals (NIH Publications No. 8023, revised 1987) and were approved by the Institutional review board of Jining Medical University (2019-YY-003).

### 2.3. Animal Models

Adult BALB/c mice weighing 14–16 g were procured from Xingkang Biotechnology Co., Ltd. (Jinan, China). The mice were allowed a period of 7 days for acclimatization prior to the commencement of the experiments. Subsequently, the mice were randomly divided into four groups, with each group consisting of five mice. A lethal dose of Pseudomonas aeruginosa ATCC 27853 (2 × 10^7^ CFU/mouse) was intraperitoneally injected into the mice to establish a bacterial infection model. Gj-CATH5 (10 mg/kg) in PBS was administered either 24 h before (−24 h) or 1 h after (+1 h) infection. After an additional 18 h, peritoneal lavage fluids and spleen samples were collected and subjected to viable bacteria assessment and ELISA assay.

### 2.4. In Vivo Neutrophil and Macrophage Recruitment

In this study, a dosage of 10 mg/kg of Gj-CATH5 in PBS was administered via injection into the peritoneal cavity of BALB/c mice. After a 24 h period, the peritoneal lavage was collected and the total cells were stained using APC-labeled anti-mouse F4/80 recombinant antibody and FITC-labeled anti-mouse Ly-6G/Ly-6C (Gr-1) antibody from Biolegend, SanDiego, CA, USA. This staining process was conducted for a duration of 30 min at room temperature. The flow cytometry technique, specifically utilizing the CytoFlex instrument (Beckman, Brea, CA, USA), was employed to determine the percentage of neutrophils and macrophages present in the peritoneal exudate cells.

### 2.5. Measurement of Intracellular Reactive Oxygen Species

Intracellular reactive oxygen species (ROS) generation was assessed following exposure to varying concentrations of Gj-CATH5 (2, 4, and 8 µg/mL) using the Reactive Oxygen Species Assay Kit (Beyotime Biotechnology, Shanghai, China). Cells were diluted to a concentration of 1 × 10^6^ cells/mL in RPMI 1640 and seeded in 24-well culture plates with a volume of 500 µL per well. After adherence, the cells were stimulated with Gj-CATH5 for 24 h, washed three times with PBS, and then incubated with 10 µmoL DCFH-DA for 20 min at 37 °C. The intracellular fluorescence was quantified using a CytoFlex flow cytometer (Beckman, Brea, CA, USA) with an excitation wavelength of 488 nm and an emission wavelength of 525 nm.

### 2.6. Phagocytosis Assay

*Pseudomonas aeruginosa* ATCC 27853 was diluted to a concentration of 5 × 10^8^ colony-forming units per milliliter (CFU/mL) using a carbonate buffer solution. The bacteria were then loaded with a FITC fluorescent dye (Sigma, Livonia, MI, USA) at a concentration of 0.3 mg/mL for a duration of 2 h at a temperature of 37 °C. Following this, the bacteria were washed three times with PBS and subsequently fixed with a 1% paraformaldehyde solution for a period of 30 min. The fixed bacteria were then washed twice with fresh PBS. J774A.1 cells were pre-treated with varying concentrations (ranging from 0 to 8 µg/mL) of Gj-CATH5 for a duration of 18 h. Subsequently, the cells were incubated with the FITC-labelled *P. aeruginosa* ATCC 27853 at a multiplicity of 10 for a period of 2 h at a temperature of 37 °C. After washing the cells with ice-cold PBS, the percentage and mean fluorescence intensity (MFI) of intracellular FITC-labelled strains were determined using a CytoFlex flow cytometer (Beckman, Brea, CA, USA). Cells that were not incubated with FITC-labelled strain were used as background.

### 2.7. Bacterial Killing Assay

The bacterial killing assay was conducted according to previously established protocols [8,15]. PMN and J774A.1 cells were cultured in 24-well plates and exposed to varying concentrations (0–16 µg/mL) of Gj-CATH5. After a specified time interval, serum-opsonized *P. aeruginosa* ATCC 27853 at a multiplicity of infection (MOI) of 5 was introduced into the cell medium, followed by a 60 min co-incubation period at 37 °C. Subsequently, CFUs were quantified by plating 10-fold serial dilutions of cell lysates on LB agar plates and incubating them at 37 °C for 18 h. The percentage of bacterial killing was calculated as [1 − (CFU_treatment_/CFU_bacteria-only controls_)] × 100%.

### 2.8. Myeloperoxidase (MPO) Activity Assay

PMN cells were resuspended in RPMI 1640 medium and subsequently seeded in a 24-well plate at a concentration of 5 × 10^4^ cells/mL. Various concentrations of Gj-CATH5 (ranging from 0 to 16 µg/mL) were added to the cell medium, followed by a 2 h incubation period. The resulting cell pellet was obtained through centrifugation (5 min, 1100× *g*, 4 °C). Intracellular MPO activity was determined using the MPO Activity assay kit from Elabscience, Wuhan, China, following the manufacturer’s instructions. MPO activity was expressed in units per milligram of neutrophils.

### 2.9. NETs Entrapment Assay

PMN were pre-stimulated with 15 µg/mL of phorbol 12-myristate 13-acetate (PMA) for 20 min. Gj-CATH5 peptides were subsequently added at concentrations ranging from 0 to 16 µg/mL, followed by a 30-min incubation at 37 °C. Subsequently, the cells were infected with FITC-labeled Pseudomonas aeruginosa ATCC 27853 at an MOI of 200 and incubated for 90 min. After three washes, the fluorescence values of the plates were measured at 485/525 nm using the SuPerMax 3100 multi-mode microplate reader (Flash, Beijing, China). The percentage of entrapment was calculated by comparing the total bacteria fluorescence of the group without PMN.

### 2.10. Minimum Bactericidal Concentration (MBC) Assay

The broth microdilution approach was employed to perform the MBC assay as described by the Clinical and Laboratory Standards Institute (CLSI). *Streptococcus. mutans* UA 159 cells were routinely cultivated in brain heart infusion (BHI) broth (Oxoid, Hampshire, UK) and incubated anaerobically (85% N_2_, 10% H_2_, and 5% CO_2_) at 37 °C. The other strains, including *Escherichia coli* ATCC25922, *Pseudomonas aeruginosa* CMCC10104, *P. aeruginosa* ATCC 27853, *Klebsiella pneumoniae* CMCC46117, *K. pneumonia* IS1368, *Staphylococcus aureus* CMCC26003, and *Staphylococcus epidermidis* CMCC26069, were grown in cation-adjusted Mueller–Hinton broth (CAMHB) medium with aeration at 35 ± 2 °C. All strains were cultivated until they reached the logarithmic growth phase. Subsequently, the cells were diluted to a concentration of 2 × 10^5^ cells/mL for experimental purposes. Serial dilutions of Gj-CATH5 were prepared in 96-well microtiter plates, followed by the addition of equivalent volumes of bacterial suspensions. The plates were then incubated under anaerobic conditions at 37 °C for *S. mutans* UA159, or at 35 ± 2 °C with aeration for the other tested strains, for a duration of 16–18 h. The minimum concentrations at which no bacterial growth was observed were recorded as the MBC values. Cephalexin and amikacin sulfate were used as control substances. All MBC values were reported in μg/mL in at least three independent experiments.

### 2.11. Cytotoxicity and Hemolysis Assays

The cytotoxicities of Gj-CATH5 against mouse macrophages RAW264.7 and human acute myeloid leukemia cells HL-60 were assessed using the CCK-8 assay, as previously described [8]. The hemolytic activity of Gj-CATH2 was determined using freshly prepared sheep erythrocytes. Sterile saline was used as the blank control, while 1% Triton X-100 (*v*/*v*) was used as the positive control. The percentage of hemolysis was calculated using a specific formula: [(A_sample_ − A_blank_)/(A_positive_ − A_blank_)] × 100%. Each experiment was independently replicated three times.

### 2.12. Structure Analysis

The amphipathicity of the peptide was analyzed by plotting the helical wheel diagrams online (https://heliquest.ipmc.cnrs.fr/cgi-bin/ComputParams.py (accessed on 14 October 2021)). The 3D structure of Gj-CATH5 was built using C-QUARK ab initio software (version 2021) [8]. The generated three-dimensional structure was then visualized by PyMol version 3.1.1 software.

### 2.13. Statistical Analysis

All statistical analyses were performed using GraphPad Prism V.5.0 software (GraphPad Software Inc., SanDiego, CA, USA). Unpaired Student’s *t*-tests were conducted to compare the two groups. Two-tailed *p* < 0.05 (*) and *p* < 0.01 (**) signified statistical significance.

## 3. Results

### 3.1. Efficacy of Gj-CATH5 Against Pseudomonas aeruginosa Infection

**Figure 1:**
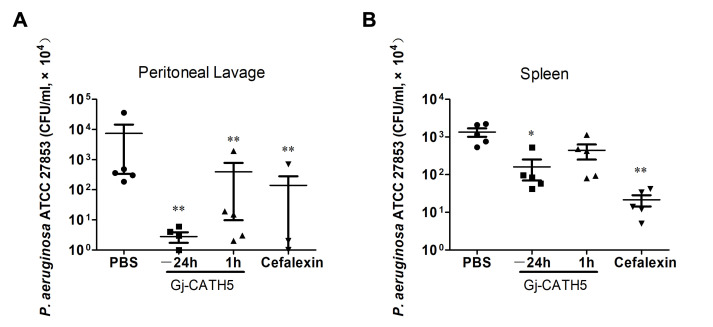
Efficacy of Gj-CATH5 in the *P. aeruginosa* infection model. At the indicated times before (−) or after (+) bacterial infection, Gj-CATH5 was administered by i.p. injection. Bacterial counts in mice peritoneal lavage (**A**) and spleens (**B**) were then assessed. *, *p* < 0.05; **, *p* < 0.01; by unpaired *t* test.

In order to assess the efficacy of Gj-CATH5 as an anti-infective agent, a mouse model of systemic infection with *P. aeruginosa* was utilized. The results demonstrated that Gj-CATH5 provided robust protection against infection, regardless of whether it was administered 24 h prior to infection or 1 h after (Figure 1). Notably, Gj-CATH5 significantly decreased the number of viable bacteria in both the peritoneal lavage fluid and the spleen of the mice. Furthermore, the pretreatment administration group (−24 h) exhibited a more pronounced therapeutic effect compared to the post-infection administration group (+1 h), suggesting the potential immunomodulatory properties of Gj-CATH5 in the context of anti-infection therapy. *P. aeruginosa* is a common opportunistic pathogen in the skin, respiratory tract, and digestive tract of humans and farm animals [16]. More research is needed to further explore the effect of Gj-CATH5 in pneumonia caused by *P. aeruginosa* infection.

**Figure 2:**
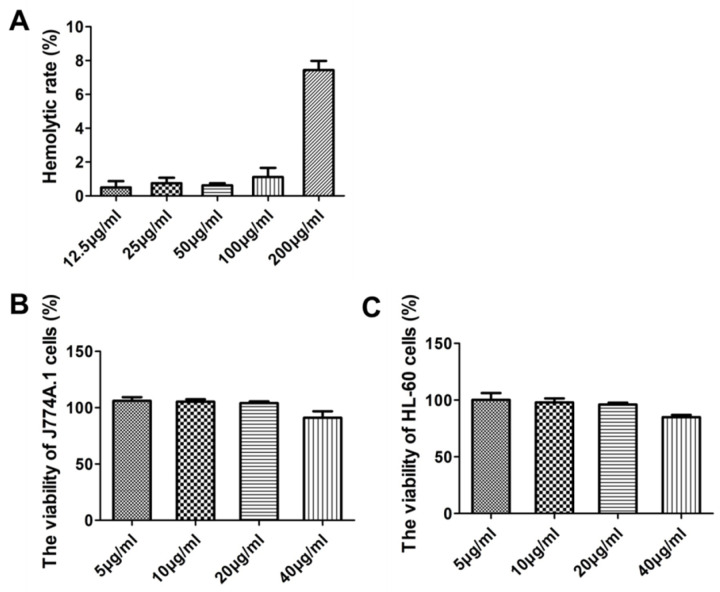
Hemolysis and cytotoxicity of Gj-CATH5. (**A**) Hemolysis of Gj-CATH5 against sheep erythrocytes. (**B**,**C**) Cytotoxicity of Gj-CATH5 against J774A.1 and HL-60.

The hemolysis and cytotoxicity of Gj-CATH5 on normal cells were examined in this study. Figure 2A demonstrates that even at an action concentration of up to 200 μg/mL, the hemolysis rate of Gj-CATH5 and its modifications on sheep red blood cells remained below 8%. Furthermore, the CCK-8 method was employed to assess the cytotoxicity of Gj-CATH5 on J774A.1 and HL-60 cells. Figure 2B,C illustrates that at an active concentration of 20 μg/mL, Gj-CATH5 exhibited no significant cytotoxicity towards both cell types. However, a slight decrease in the survival rate of both cells was observed when the concentration of Gj-CATH5 was increased to 40 μg/mL.

### 3.2. Gj-CATH5 Exhibited a Significant Chemotactic Effect on Neutrophils and Monocytes/Macrophages

**Figure 3:**
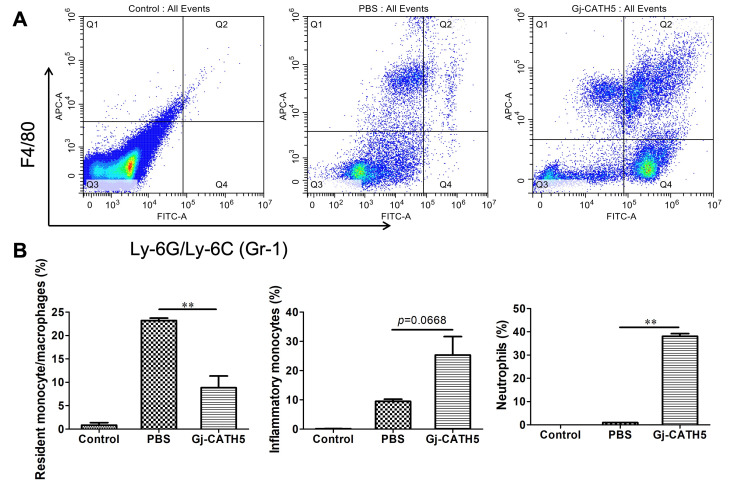
Gj-CATH5 regulates macrophage/monocyte and neutrophil cell trafficking in vivo. (**A**) Total cells in peritoneal lavage were collected, stained, and analyzed by flow cytometry 24 h after Gj-CATH5 administration. Resident monocytes/macrophages, inflammatory monocytes, and neutrophils were gated as GR1−/F4/80+, GR1+/F4/80+, and GR1+/F4/80−, respectively. (**B**) The number of resident monocytes/macrophages, inflammatory monocytes, and neutrophils was counted after Gj-CATH5 administration. The results are reported as mean ± SEM from three mice (**, *p* < 0.01; by unpaired *t* test). Control, untreated mice group; PBS, mice treated with PBS; Gj-CATH5, mice treated with 10 mg/kg of Gj-CATH5 in PBS.

Macrophages and neutrophils are crucial effector cells in the innate immune system, playing a vital role in the host’s defense against extracellular pathogen infection and acute inflammation. In order to ascertain the contribution of these innate immune cells to the anti-infective activity of Gj-CATH5, the chemotactic response of these cells to Gj-CATH5 was initially assessed. Figure 3 demonstrates that Gj-CATH5 effectively induced chemotaxis of neutrophils and monocytes following 24 h of treatment and prompted the differentiation of resident monocytes/macrophages into inflammatory monocytes. Following Gj-CATH5 treatment, the population of resident monocytes/macrophages decreased from 23.20% to 8.85% compared to the PBS group, while there was a slight increase in the number of inflammatory monocytes (*p* = 0.0668). Conversely, the chemotactic impact of Gj-CATH5 on neutrophils was more pronounced, with nearly 40% of neutrophils present in the abdominal cavity of mice after Gj-CATH5 injection (Figure 3B).

### 3.3. Gj-CATH5 Has a Regulatory Effect on the Phagocytic Bactericidal Activity of Macrophages

**Figure 4:**
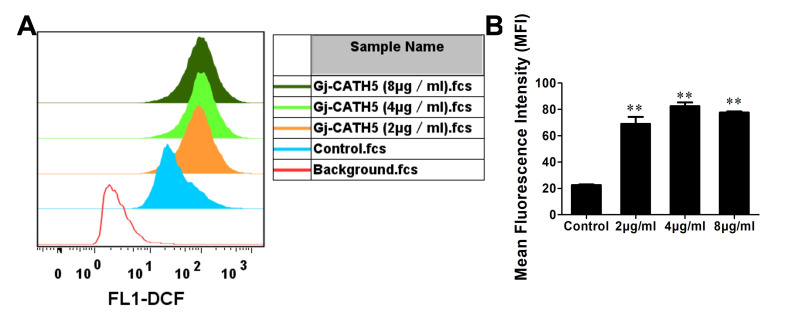
Effects of Gj-CATH5 on ROS production of J774A.1 cells. (**A**) Gj-CATH5 effectively enhanced the intracellular ROS production in J774A.1 cells. (**B**) The MFI of J774A.1 cells in (**A**). The results are reported as mean ± SD in three independent experiments (**, *p* < 0.01; by unpaired *t* test).

We investigated the impact of Gj-CATH5 on the production of reactive oxygen species (ROS) in J774A.1 cells, which are a type of immune cell involved in phagocytic bactericidal function. Our results, as depicted in Figure 4A, demonstrate that Gj-CATH5 effectively enhanced the intracellular ROS production in J774A.1 cells in a concentration-dependent manner. Additionally, the MFI of J774A.1 cells was significantly elevated in response to Gj-CATH5 (Figure 4B).

**Figure 5:**
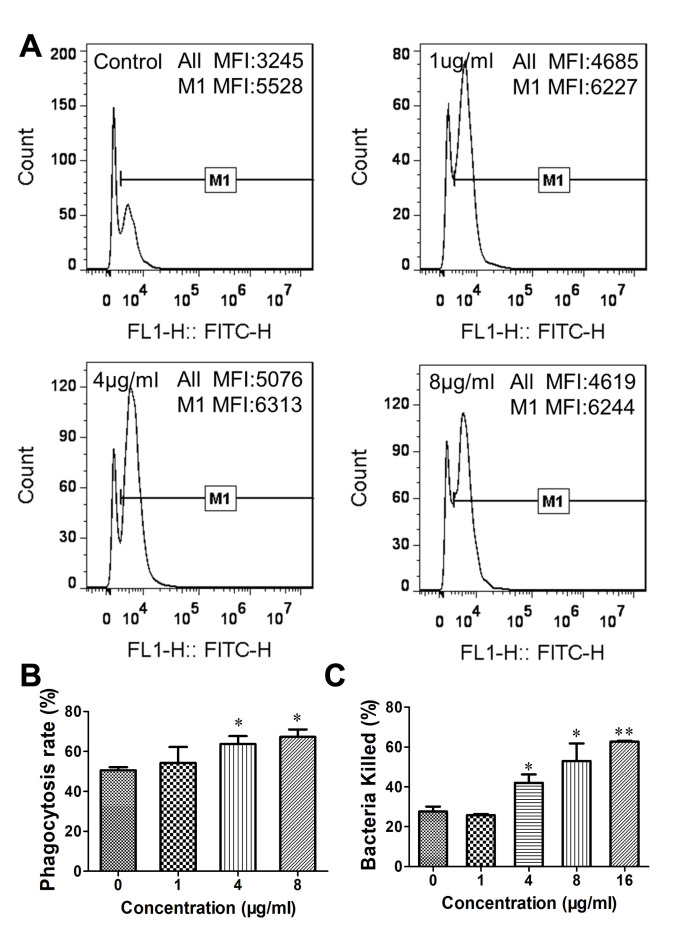
Effects of Gj-CATH5 on the phagocytosis (**A**,**B**) and bacteria-killing capacity (**C**) of J774A.1 cells. Data are expressed as mean ± SEM from three independent experiments. *, *p* < 0.05; **, *p* < 0.01; by unpaired *t* test.

In addition, the impact of Gj-CATH5 on the phagocytosis activity of macrophages was investigated. The results depicted in Figure 5A,B revealed that a high concentration of Gj-CATH5 (≥4 μg/mL) significantly enhanced the phagocytosis rate of J774A.1 cells against *P. aeruginosa* (the positive rate of cells indicated the phagocytosis rate). Following pre-treatment with 8 μg/mL of Gj-CATH5, the phagocytosis rate of J774A.1 against *P. aeruginosa* escalated from 50.57% to 67.3%. Furthermore, the average fluorescence intensity of the positive cells exhibited an increase when compared to the control group. These findings demonstrate that pretreatment with Gj-CATH5 enhances macrophage phagocytosis of pathogens and also increases the number of pathogens phagocytosed by individual macrophages. Additionally, the results of the bactericidal efficiency experiment provide further evidence that Gj-CATH5 pretreatment significantly enhances the bactericidal activity of macrophages against *P. aeruginosa* in a concentration-dependent manner (Figure 5C). Specifically, the bactericidal efficiency of J774A.1 cells against pathogens increased from 27.6% to 42.1%, 53.0%, and 62.7% under the concentration of 4, 8, and 16 μg/mL of Gj-CATH5, respectively.

### 3.4. Gj-CATH5 Regulates the Bactericidal Function of Neutrophil

**Figure 6:**
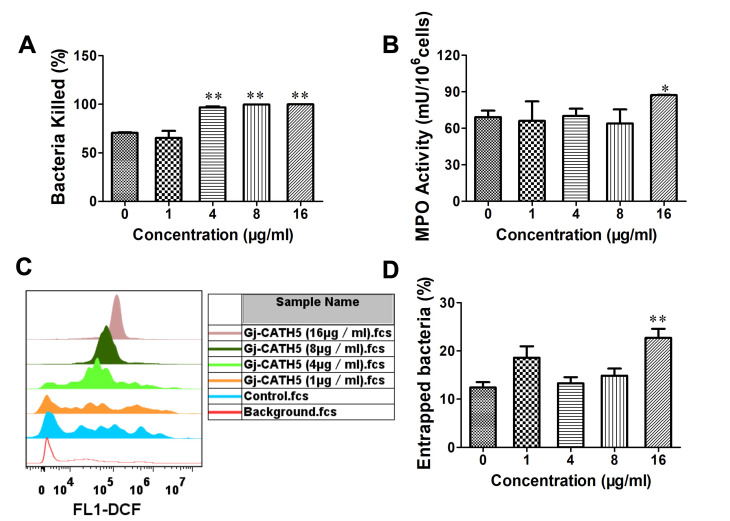
Effects of Gj-CATH5 on the bactericidal function of neutrophils. (**A**) Gj-CATH5 of various concentrations potentiated the bacterial killing capacity of neutrophils. (**B**) Gj-CATH5 in high concentration increased the intracellular MPO activity of neutrophils. (**C**,**D**) Effects of Gj-CATH5 on entrapment of *P. aeruginosa* ATCC 27853 by NETs-releasing neutrophils. Data are expressed as mean ± SEM from at least three independent experiments. *, *p* < 0.05; **, *p* < 0.01; by unpaired *t* test.

We conducted an investigation on the impact of Gj-CATH5 on neutrophil-mediated bactericidal activity. The results presented in Figure 6A demonstrate that the pre-treatment of neutrophils with Gj-CATH5 significantly enhanced their ability to kill bacteria, with the effectiveness being dependent on both the concentration and duration of treatment. Specifically, neutrophils treated with 4 μg/mL of Gj-CATH5 were able to eliminate 96% of *P. aeruginosa* within 1 h (Figure 6A). Following the recognition of invading pathogens by neutrophils, phagocytosis occurs within the phagocytic body, leading to the elimination of the pathogens through both oxygen-dependent mechanisms involving reactive oxygen species and oxygen-independent mechanisms such as intracellular degranulation. In order to further explore the mechanism by which Gj-CATH5 enhances the bactericidal activity of neutrophils, we examined the effect of Gj-CATH5 on the production of ROS in neutrophils. The results showed that Gj-CATH5 pretreatment significantly upregulated the production of ROS in neutrophils (Figure 6C). MPO is a marker of neutrophil function and activation, and the peroxidase activity of MPO generates hypochlorite to kill microbes and inactivate inhibitors of lytic enzymes. High concentration of Gj-CATH5 also promoted and increased the intracellular MPO activity of neutrophils (Figure 6B).

Furthermore, aside from phagocytosis and degranulation, neutrophils have been discovered to employ an indirect method of microorganism eradication by releasing their intracellular material to form a structure known as Neutrophil Extracellular Traps (NETs) [17]. We investigate the impact of Gj-CATH5 on the efficacy of NETs in capturing pathogens. The results, as depicted in Figure 6D, indicate a significant increase in the number of pathogens ensnared by NETs in the 16 μg/mL Gj-CATH5 treatment group compared to the control group, nearly doubling the amount of trapped pathogens. To determine whether Gj-CATH5 is a direct driver of neutrophil extracellular trap (NET) formation and reactive oxygen species (ROS) production, we set a negative control group in this study, where neutrophils are not exposed to Gj-CATH5. This group helps establish the baseline level of NET formation and ROS production in the absence of the peptide, thereby allowing for a comparison with the experimental group. However, to confirm the problem that Gj-CATH5 is the direct rather than secondary driver of infection or inflammation, a positive control using a known inducer of NET formation, such as phorbol 12-myristate 13-acetate (PMA), should be included to confirm the neutrophils’ capacity to form NETs under stimulatory conditions in a further study. Furthermore, to rule out the possibility that Gj-CATH5’s effects are secondary to infection or inflammation, it would be beneficial to include a control group where neutrophils are exposed to an inflammatory stimulus, such as lipopolysaccharide (LPS), without Gj-CATH5. This would help differentiate between the effects of Gj-CATH5 and those of general inflammatory stimuli. Additionally, the use of specific inhibitors of ROS production, such as N-acetylcysteine (NAC), could help determine whether ROS production is a necessary component of Gj-CATH5-induced NET formation.

### 3.5. The Direct Bactericidal Activity of Gj-CATH5

**Figure 7:**
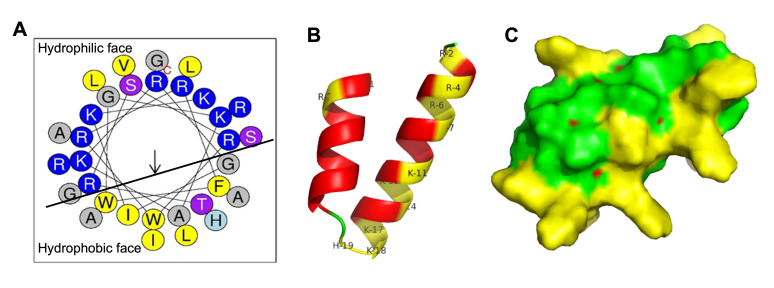
Structure characterizations of Gj-CATH5. (**A**) Helix-wheel plots of Gj-CATH5. The hydrophobic and hydrophilic faces are separated by a line. (**B**,**C**) Tertiary structures of Gj-CATH5. Surface representation of Gj-CATH5 is shown in green with the positively charged residues in yellow.

The findings from the antibacterial experiments indicate that Gj-CATH5 exhibits broad-spectrum and highly effective bactericidal properties, particularly against antibiotic-resistant bacteria. The MBC of Gj-CATH5 ranges mostly between 4 and 8 µg/mL, as presented in Table 1. In general, Gj-CATH5 demonstrates superior antimicrobial activity against Gram-negative bacteria compared to Gram-positive bacteria, potentially due to the impact of the thicker peptidoglycan structure in Gram-positive cell walls on Gj-CATH5’s cellular penetration ability. Existing literature extensively documents the ability of cathelicidin family antimicrobial peptides to target bacterial cell membranes [18,19]. Moreover, in conjunction with the distinct bactericidal impact of Gj-CATH5 on both gram-positive and gram-negative bacteria, Gj-CATH5 has the potential to penetrate bacterial cells and exhibit bactericidal properties by influencing the synthesis of nucleic acids or proteins within these cells. This aspect merits further investigation and scholarly inquiry. Structural examination has revealed that Gj-CATH5 predominantly adopts an amphipathic α-helical conformation with an overall positive charge (as depicted in Figure 7). This structural attribute may enhance its antibacterial efficacy when interacting with microbial cell plasma membranes or toxin constituents.

## 4. Discussion

Cathelicidins have garnered significant attention as potential therapeutic agents due to their unique mechanism of action and potent antibacterial activity. These small, cationic peptides are part of the innate immune system and are found in humans and other species, including farm animals. They exhibit a broad spectrum of antimicrobial activity against bacteria, enveloped viruses, and fungi, making them versatile agents in combating infections [20]. This article reports the mechanism of gecko cathelicidin Gj-CATH5 in regulating immune cells to combat *P. aeruginosa* infection. This bacterium is known for its ability to cause severe infections, which are often difficult to treat due to its resistance to many antibiotics and its ability to form biofilms. Our study found that Gj-CATH5 orchestrates the recruitment and activation of macrophages and neutrophils to the site of *P. aeruginosa* infection. Pattern recognition receptors (PRRs) play a crucial role in the innate immune system by recognizing pathogen-associated molecular patterns (PAMPs) and damage-associated molecular patterns (DAMPs), thereby initiating immune responses. IDRs can stimulate pattern recognition receptors (PRRs) on innate immune cells [21]. For instance, cathelicidin has been shown to boost the antifungal activity of neutrophils during Aspergillus fumigatus keratitis. This is achieved by promoting neutrophil phagocytosis and degradation of fungal conidia, which is mediated through the activation of the CXC chemokine receptor 2 (CXCR2) and the induction of autophagy [22]. Understanding the mechanism by which PRRs, such as cathelicidin Gj-CATH5, activate macrophages and neutrophils is essential for elucidating their role in immune defense and potential therapeutic applications. Macrophages and neutrophils are key players in the innate immune response, with PRRs expressed on their surfaces facilitating the detection of pathogens. In the context of macrophages, PRRs such as Toll-like receptors (TLRs) and C-type lectin receptors (CLRs) are known to recognize microbial components, leading to the activation of signaling pathways that result in the production of cytokines and chemokines, which are crucial for orchestrating the immune response [21]. Similarly, neutrophils, which were once thought to be transcriptionally inert, have been shown to express a broad repertoire of PRRs. These receptors enable neutrophils to respond dynamically to infections, thereby playing a significant role in both protective and pathogenic immune responses [23]. The activation of macrophages and neutrophils by PRRs involves complex signaling pathways. For instance, in the case of neutrophils, G protein-coupled receptors (GPCRs) are involved in recognizing diverse ligands, leading to various downstream effects such as cell recruitment to sites of inflammation and modulation of inflammatory processes [24]. This highlights the importance of PRRs in regulating immune cell functions and their potential as targets for therapeutic interventions. Further study is needed to discover the mechanism by which Gj-CATH5 activates macrophages and neutrophils.

Gj-CATH5 can also modulate the function of immune cells. Under the treatment of high concentration Gj-CATH5, the NETs entrapment of *P. aeruginosa* by neutrophils was significantly improved. Neutrophils are crucial components of the innate immune system, and their ability to combat infections is significantly enhanced by the formation of NETs, which trap and kill pathogens. NETs consist of decondensed chromatin fibers decorated with antimicrobial proteins, and their formation is a critical response to bacterial infections, including those caused by *P. aeruginosa* [25]. *Pseudomonas aeruginosa* is known for its ability to form biofilms, which are resistant to both immune responses and antibiotic treatments. The biofilm matrix is composed of extracellular polymeric substances, including DNA, which can be released by the bacteria themselves or derived from host cells such as neutrophils through NETosis [26]. The interaction between NETs and *P. aeruginosa* biofilms is complex, as NETs can both trap bacteria and contribute to the inflammatory response, potentially exacerbating tissue damage [27]. The antimicrobial activity of NETs is not solely dependent on the proteins they contain; the DNA backbone itself has been shown to possess bactericidal properties by sequestering cations and disrupting bacterial membranes [28]. However, *P. aeruginosa* has developed mechanisms to resist NET-mediated killing, such as the expression of surface modifications that protect against DNA-induced membrane destabilization [28].

In addition to NETs, neutrophils and macrophages play a vital role in the immune response against *P. aeruginosa* by phagocytosing and killing bacteria. However, *P. aeruginosa* can evade macrophage-mediated killing by promoting autophagy, which suppresses phagocytosis and intracellular killing [29]. This evasion strategy highlights the importance of enhancing macrophage function to effectively combat *P. aeruginosa* infections. The modulation of immune responses by antimicrobial peptides like Gj-CATH5 could potentially enhance the effectiveness of both neutrophils and macrophages. Cathelicidins, a family of antimicrobial peptides, are known to have immunomodulatory effects, including the enhancement of phagocytosis and the modulation of cytokine production [30]. By influencing these processes, Gj-CATH5 may improve the ability of neutrophils and macrophages to trap and kill *P. aeruginosa*, thereby providing a more robust defense against this opportunistic pathogen. Overall, the interplay between NETs, macrophages, and antimicrobial peptides like Gj-CATH5 is crucial in the defense against *P. aeruginosa*. Understanding these interactions can lead to the development of novel therapeutic strategies that enhance the innate immune response and improve outcomes in infections caused by this challenging pathogen.

**Figure 8:**
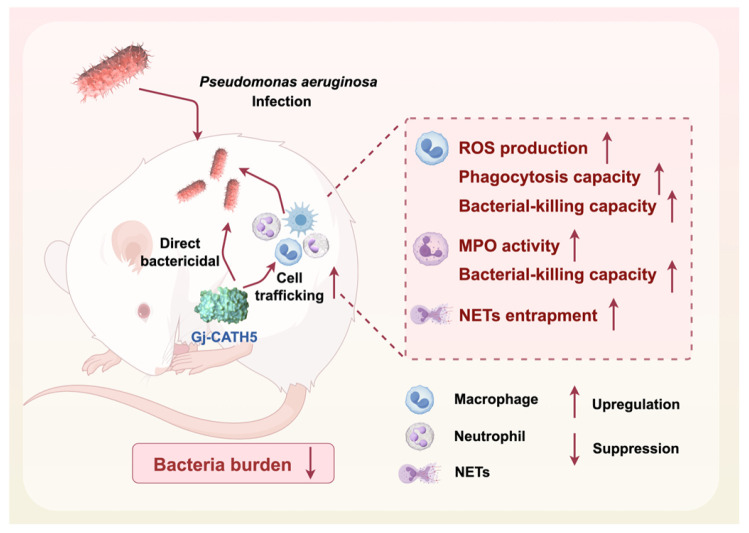
Graphical summary of the study. The figure was created with Figdraw.

Furthermore, the gecko cathelicidin Gj-CATH5 exhibits a unique dual mechanism in combating *P. aeruginosa* infections (Figure 8), which is notably innovative compared to other reported antimicrobial peptides. Firstly, Gj-CATH5 effectively inhibits the growth of Pseudomonas aeruginosa through direct bactericidal action. This bactericidal effect is similar to that of other known antimicrobial peptides, such as Bac7 (1-35), which kill bacteria by disrupting their cell membranes [31]. Furthermore, Gj-CATH5 enhances the host’s ability to fight infections by regulating the function of immune cells. Specifically, it boosts the phagocytic activity of macrophages and neutrophils, thereby improving their efficiency in clearing bacteria. This immune modulation mechanism is similar to that of other antimicrobial peptides like Reg4, which not only directly binds to bacterial cell walls to kill bacteria but also enhances the phagocytic capacity of alveolar macrophages in the host [32]. In contrast, other antimicrobial peptides like LL-37 primarily prevent bacterial invasion by increasing the stiffness of epithelial cells and reducing cell permeability [33]. The innovative aspect of Gj-CATH5 is that it not only has direct bactericidal capabilities but also provides comprehensive anti-infection protection by regulating the function of immune cells. This dual mechanism makes Gj-CATH5 more promising and applicable in combating infections caused by multidrug-resistant strains.

## 5. Conclusions

This study investigated the protective efficacy and underlying mechanisms of Gj-CATH5, a novel cathelicidin derived from *G. japonicus*, in combating *P. aeruginosa* infections. Both in vitro and in vivo experiments demonstrated that Gj-CATH5 significantly reduced bacterial load, enhanced the recruitment and activation of neutrophils and macrophages, and facilitated the production of reactive oxygen species, myeloperoxidase activity, and neutrophil extracellular trap formation (Figure 8). The amphipathic α-helical structure of Gj-CATH5 conferred broad-spectrum bactericidal properties and low cytotoxicity. Collectively, Gj-CATH5 exhibited a dual function in direct pathogen eradication and immune modulation, indicating its potential as a promising candidate for addressing multidrug-resistant infections and laying the groundwork for the development of peptide-based immune therapeutics targeting challenging bacterial pathogens.

## Figures and Tables

**Table 1:** The bactericidal activity of Gj-CATH5.

Microorganism Strains	MBC ^1^ (µg/mL)		
	Gj-CATH5	Cephalexin	Amikacin Sulfate
Gram-negative bacteria			
*Escherichia coli* ATCC25922	8	16	8
*Pseudomonas aeruginosa* CMCC10104	8	-	16
*P. aeruginosa* ATCC 27853	8	-	16
*Klebsiella pneumonia* CMCC46117	-	-	16
*K. pneumonia* IS1368	8	16	32
Gram-positive bacteria			
*Staphylococcus aureus* CMCC26003	8	4	4
*Staphylococcus epidermidis* CMCC26069	64	4	8
*Streptococcus mutans* UA159	4	-	16

^1^ MBC: minimum bactericidal concentration.

## Data Availability

The original contributions presented in this study are included in the article. Further inquiries can be directed to the corresponding authors.

## Abbreviations

The following abbreviations are used in this manuscript:

PMN	Primary mouse bone marrow-derived neutrophils
CFU	Colony-forming units
MFI	Mean fluorescence intensity
MOI	Multiplicity of infection
MPO	Myeloperoxidase
PMA	Phorbol 12-myristate 13-acetate
MBC	Minimum bactericidal concentration
NETs	Neutrophil Extracellular Traps
